# A hair-follicle reconstructed in vitro immunocompetent skin model for prediction of the sensitizing potential of chemicals

**DOI:** 10.1007/s00204-025-04130-z

**Published:** 2025-07-18

**Authors:** Tarada Tripetchr, Marla Dubau, Sarah Hedtrich, Burkhard Kleuser

**Affiliations:** 1 Institute of Pharmacy, Department of Pharmacology and Toxicology, Freie Universität Berlin, Königin-Luise-Str. 2+4, 14195 Berlin, Germany; 2 Faculty of Pharmaceutical Sciences, University of British Columbia, Vancouver, BC Canada; 3 Center of Biological Design, Berlin Institute of Health at Charité–Universitätsmedizin, Berlin, Germany; 4 School of Biomedical Engineering, University of British Columbia, Vancouver, BC Canada; 5 Centre for Blood Research & Life Science Institute, Life Science Centre, University of British Columbia, Vancouver, BC Canada

**Keywords:** Hair follicles, Keratinocytes, Fibroblasts, Dendritic cells, T-lymphocytes, Skin sensitization

## Abstract

The development of immunocompetent skin models represents a significant advancement in in vitro methods for detecting skin sensitizers, adhering to the 3R principles aimed at reducing, refining and replacing animal testing. In the present study, an advanced skin model from hair follicle-derived cells was constructed and enriched with two key immune cell types, namely Langerhans cells and T-lymphocytes, named ImmuSkin-MT. The model features a physiologically relevant epidermis and dermis, integration of monocyte-derived Langerhans cells (MoLCs) beneath the dermal layer, and co-cultivation with CD4^+^-T cells in the lower chamber of a transwell system. This setup closely mimics the native interplay between skin-resident immune cells and T-cells, marking a significant advancement in in vitro toxicology. When exposed to known sensitizers of varying potency, the model demonstrated a robust ability to predict the sensitizing potential of chemicals. By addressing different key events in skin sensitization, a differentiation between extreme, moderate and even weak sensitizers was achieved. The results showed that the MoLCs migrated, and upregulated CD86 expression in response to contact sensitizers. Additionally, proliferation of CD4^+^ T-lymphocytes was increased in response to the treatment. These results highlight the potential of the ImmuSkin-MT construct to serve as a valuable tool for mechanistic studies and future regulatory applications in the assessment of skin sensitization.

**Supplementary Information:**

The online version contains supplementary material available at 10.1007/s00204-025-04130-z.

## Introduction

Allergic contact dermatitis is an increasingly prevalent immunologically derived skin disorder of valuable prominence in industrialized countries (Uter & Diepgen 2020). It is a type IV hypersensitivity reaction that develops after allergen contact showing symptoms like itching, redness, blistering, and peeling of the skin, and therefore significantly impairs the patient’s quality of life (Di Agosta et al. 2021; Kalboussi et al. 2019). Multiple xenobiotics have the potential to trigger contact allergies, including preservatives, drugs, fragrances, and chemicals (Gerberick et al. 2008). The most effective measure to prevent the disease is exposure prophylaxis via avoiding exposure to the relevant substances. This, however implies that the skin sensitizing potential of a particular substance is known, highlighting the toxicological relevance of skin sensitization testing.

To facilitate the development of non-animal alternatives for hazard identification, the Organization for Economic Co-operation and Development (OECD) proposed an adverse outcome pathway (AOP) describing the molecular and cellular mechanisms underlying skin sensitization (OECD 2014). The AOP consists of four well-characterized key events. The molecular initiating event (Key Event 1) involves the covalent binding of a molecule (= hapten) to skin proteins, particularly at cysteine and/or lysine residues. This triggers the activation of keratinocytes (Key Event 2), which respond by releasing danger signals and pro-inflammatory mediators. Subsequently, dendritic cells (DCs) are activated (Key Event 3), a process triggered by both hapten-protein complexes and signals derived from keratinocytes. Activated DCs then migrate to adjacent lymph nodes, where they present hapten-peptide complexes to naive T-lymphocytes via major histocompatibility complex molecules, leading to the differentiation and proliferation of allergen-specific memory T-cells (Key Event 4), which elicit an inflammatory response upon re-exposure.

The murine local lymph node assay (LLNA) has long been considered as the gold standard for evaluating skin sensitization (Ahmed et al. 2016; OECD 2010). However, the implementation of the 7th Amendment to the EU Cosmetics Directive, which prohibits animal testing for cosmetic products and their ingredients, has prompted a significant shift toward the development and regulatory acceptance of alternative methods (European Union 2003).

In response, the OECD has incorporated validated in vitro and *in chemico* assays into its skin sensitization test guidelines. Notable examples are the Direct Peptide Reactivity Assay (DPRA), KeratinoSens™, and the human Cell Line Activation Test (h-CLAT), each addressing specific key events of the AOP. However, a major limitation of these methods is that they typically capture only a single key event. As a result, individual assays cannot fully replicate the complex, multicellular interactions involved in the development of contact allergy. Hence, multiple assays often need to be combined to allow a more comprehensive assessment of a chemical’s sensitizing potential (Bauch et al. 2011).

To address these limitations, various co-culture models integrating keratinocytes and immune cells such as antigen-presenting cells or lymphocytes have been developed (Frombach et al. 2018; Ouwehand et al. 2011; Sonnenburg et al. 2015; Van Den Bogaard et al. 2014). One promising approach is the loose-fit coculture-based sensitization assay (LCSA), which utilizes a co-culture of primary keratinocytes and peripheral blood mononuclear cells (PBMCs) to capture immune cell activation (Frombach et al. 2018; Sonnenburg et al. 2015). While such models offer improved biological relevance compared to single-cell assays, they still do not capture the full cascade of the AOP and often address only one key event.

Furthermore, these co-culture assays lack the structural complexity of native human skin, an essential factor for processes like cutaneous penetration and xenobiotic biotransformation. As a result, there is a growing demand for immunocompetent skin models that integrate both structural and immune components of the skin in a physiologically relevant 3D context. These advanced models provide a promising platform for assessing multiple key events simultaneously, thereby improving the predictive power and translational value of in vitro sensitization testing.

In response to this need, several immunocompetent skin models have been developed in recent years that incorporate DCs (Bock et al. 2018; Hölken et al. 2023; Kosten et al. 2015). In parallel, other approaches have focused on integrating T-cells into reconstructed skin models (Kühbacher et al. 2017; Shin et al. 2020; Wallmeyer et al. 2017). However, so far, no skin model has incorporated both types of immune cells. Despite significant progress in developing alternative methods, there is currently no approved test system for skin sensitization that is based on a reconstructed epidermis with integrated DCs and T-cells. This is largely due to the high variability and limited reproducibility of outcomes in such complex models.

Aiming to close this gap, we introduce an advanced skin model constructed from hair follicle-derived cells and enriched with two key immune cell types, monocyte-derived Langerhans cells (MoLCs) and CD4⁺ T-lymphocytes. This immunocompetent model more closely mimics the physiological structure and immune function of human skin. When exposed to known skin sensitizers, the model reliably identified the skin sensitizers by effectively capturing the key events 3 and 4 of the skin sensitization AOP. These results highlight the potential of this system to serve as a valuable tool for mechanistic studies and future regulatory applications in the assessment of skin sensitization.

## Materials and methods

### Isolation and cultivation of hair follicle-derived keratinocytes and fibroblasts

Hair follicle-derived keratinocytes (HFDKs) and hair follicle-derived fibroblasts (HFDFs) were isolated as recently described (Löwa et al. 2018). Briefly, 30–35 hair follicles were collected from donors aged between 25 and 35 years old (ethical approval EA1/345/14). Following this, 10–12 hair follicles were placed on a poly-*D*-Lysine hydrobromide solution (Sigma-Aldrich, UK) coated insert membrane of a transwell plate (Corning™Costar™, Durham, USA) containing postmitotic 3T3-J2 fibroblasts on the basal side. The hair follicles were left to culture in the outer root sheath medium (ORM +) until the cells derived from the outer root sheath (ORS) covered 80% of the insert membrane area. The ORM + consists of DMEM (Gibco, Waltham, MA, USA), 21.5% Ham’s F12 Nut mix + GlutaMax™ (Gibco, Waltham, MA, USA), 10% fetal bovine serum (FBS) (Sigma-Aldrich, Munich, Germany), 10,000 IU/mL penicillin/streptomycin (Sigma-Aldrich, Munich, Germany), 0.4 µg/mL hydrocortisone (Sigma-Aldrich, Munich, Germany), 0.1 nM cholera toxin (Sigma-Aldrich, Munich, Germany), 10 ng/mL epidermal growth factor (EGF) (Sigma-Aldrich, Munich, Germany), 5 µg/mL insulin (Roche, Basel, Switzerland), 0.18 mM adenine (Sigma-Aldrich, Munich, Germany), 4 mM *L*-glutamine, and 2 nM liothyronine (Sigma-Aldrich, Munich, Germany). Cell isolation from the outgrowth of the ORS was executed using a detach-time-selection strategy. HFDFs were obtained through a 5-min treatment of the ORS with 0.05% trypsin and 0.02% EDTA (Sigma-Aldrich, USA), while HFDKs were isolated following a 10-min treatment of the ORS outgrowth with trypsin and EDTA. HFDFs were cultured on PureCol EZ gel (Merck, Darmstadt, Germany) coated cells culture vessels in fibroblast growth medium (FGM) consisting of DMEM (Sigma-Aldrich, Munich, Germany) supplemented with 10% FBS, and 100µ/mL penicillin/streptomycin. HFDKs were cultured in keratinocyte expansion medium (Promocell, Heidelberg, Germany) on PureCol EZ gel coated cell culture vessels.

### Generation and cultivation of MoLCs

Monocytes were isolated from anonymous donor buffy coat (German Red Cross, Berlin, Germany) using the adherence method and were later differentiated into monocyte-derived Langerhans cells (MoLCs). MoLCs were differentiated by culturing monocytes in DC generation medium (Promocell, Heidelberg, Germany) supplemented with 100 ng/mL GM-CSF, 20 ng/mL TGF-β, and 20 ng/mL IL-4 for 6 days. The medium replacement was done by substituting half of the existing media with freshly supplemented media every 3 days. On day 6, CD1a + MoLCs were magnetically labeled and isolated using CD1a MicroBeads, human (Miltenyi Biotech, Bergisch Gladbach, Germany).

### Generation and cultivation of T-cells

CD4^+^ naïve T-lymphocytes were isolated from PBMC using the naive CD4^+^ T-Cell Isolation Kit II, human, as recommended by the manufacturer (Miltenyi Biotech, Bergisch-Gladbach, Germany). The PBMCs were obtained from an anonymous donor buffy coat (German Red Cross, Berlin, Germany). In brief, 10^8^ PBMCs were labeled with Biotin-Antibody Cocktail II, followed by the addition of the naïve CD4^+^ T-cell Microbead Cocktail II. The cell suspension was then processed through the LS column and the cells were enriched via the magnetic cell separator (both are from Miltenyi Biotech, Bergisch-Gladbach, Germany).

### Preparation of immunocompetent skin models

To determine the impact of different cells, three immunocompetent skin models were developed, which are ImmuSkin-M, ImmuSkin-T, and ImmuSkin-MT. These ImmuSkins were generated by cultivating reconstructed human skin models (RHS) together with MoLCs and T-lymphocytes in the same system. RHS were created following a previously described method (Löwa et al. 2018). Briefly, 0.5 × 10^6^ HFDFs were suspended in PureCol EZ gel and solidified at 37 °C without CO_2_. Subsequently, HFDKs were seeded on top of the solidified dermis layer and incubated for 24 h at 37 °C with 5% CO_2_. The RHS was then cultured in an air–liquid interface method and further cultivated with a keratinocyte differentiation medium composed of DMEM, Ham’s F12 + GlutaMax™, FBS, hydrocortisone, insulin, adenine, EGF, and cholera toxin for 12 days. To assemble immunocompetent skin models with MoLCs alone (ImmuSkin-M), 0.5–1 × 10^6^ MoLCs were resuspended in PureCol EZ gel and positioned beneath a matured RHS. For the immunocompetent skin model with only naïve CD4^+^ T-lymphocytes (ImmuSkin-T), an RHS was placed on top of a solidified acellular PureCol EZ gel layer. CellTrace™ CFSE Cell Proliferation Kit was used for labeling of T-cells to trace multiple generations using dye dilution by flow cytometry as recommended by the manufacturer (Thermo Fischer Scientific, Karlsruhe, Germany). 0.5–1 × 10^6^ of labeled CD4^+^ T-lymphocytes per well were added in the lower chamber of the transwell containing DC generation medium.

To create the immunocompetent skin model containing both, MoLCs and CD4^+^ T-lymphocytes (ImmuSkin-MT), the MoLC layer was assembled similarly to ImmuSkin-M, followed by the addition of carboxyfluorescein succinimidyl ester (CFSE)-labeled naïve CD4^+^ T-lymphocytes into the lower chamber similar to ImmuSkin-T. To account for biological variation, immune responses were evaluated using three independent donor combinations, including both male and female donors aged 25–35 years.

### Skin sensitization assay

To assess the performance of the ImmuSkins in identifying skin sensitizers, xenobiotics with different skin sensitizing potential were topically applied onto the skin models. More specifically, 5 µM 2,4-dinitrochlorobenzene (DNCB) and 10 µM *p*-phenylenediamine were included as extreme skin sensitizers, 300 µM isoeugenol as a strong sensitizer, and 250 µM resorcinol as a moderate sensitizer. 500 µM glycerol was included as a non-sensitizer, and 0.1% dimethyl sulfoxide (DMSO), the solvent of the substances, served as a negative control. All the chemicals were purchased from Sigma–Aldrich (Munich, Germany). Additionally, a pro-inflammatory cytokine cocktail containing 50 ng/mL TNF-α and 50 ng/mL IL-1β served as a positive control (both are from Miltenyi Biotech, Bergisch Gladbach, Germany). The stock solution of each skin sensitizer was prepared using DMSO as a solvent and were diluted to 1:1000 in the DC generation medium, resulting in a DMSO concentration ≤ 0.1%. Untreated exposed ImmuSkin was served as untreated control. The concentrations of the individual skin sensitizers were selected based on various previous studies (Ahmed et al. 2016; D. A. Basketter et al. 2005; Ouwehand et al. 2011; Rees et al. 2011b). 24 h after topical application, the cells from the lower chamber of the transwell were collected for flow cytometry analysis. The immunocompetent skin models were embedded in FSC 22 frozen section compound (Leica Biosystem, Wetzlar, Germany) and snap frozen with liquid nitrogen for further analysis.

### Migration and maturation of epidermal MoLCs

MoLC collected from the culture medium of the lower chamber of the transwell, derived from ImmuSkin-M, ImmuSkin-T, or ImmuSkin-MT, were stained against the following extracellular markers, anti-CD1a-FITC-, anti-CD86-BV605-, APC/cyanine7 anti-CD4-antibodies (Biolegend, San Diego, USA) and 7-aminoactinomycin D (7-AAD) (StemCell Technology, Cologne, Germany) according to standard procedures. Staining was performed by washing the cells twice in Cells Staining Buffer (Biolegend, San Diego, USA) followed by an incubation with the marker cocktail for 30 min in the dark on ice. Subsequently, any excess marker was washed off, and 7-AAD was added directly prior to the analysis. Unstained cells served as a background reference. Compensation was achieved using UltraComp eBeads™ Compensation Beads (Thermo Fisher Scientific, Karlsruhe, Germany) and fluorescence signals were measured using CytoFLEX (Beckman Coulter, Krefeld, Germany). Median fluorescence intensity (MFI) and cell frequency were analyzed using FlowJo version 10.8.01 (BD Bioscience, San Jose, USA). MoLC migration rate was determined by number of CD1a + cells found in the lower chamber using flow cytometry, while MoLC maturation was measured by the MFI of CD86 of the live CD1a + population.

### Proliferation of T-cells

To assess the proliferative capacity of T-cells into the immunocompetent skin models, CellTrace™ CFSE Cell Proliferation Kits were used to tag CD4^+^ T-lymphocytes according to the manufacturer's instructions. Briefly, 1 × 10^6^ T-lymphocytes were resuspended in 1 mL PBS and mixed with 1µL of 5 mM CellTrace™ CFSE dye. The cell suspension was incubated for 20 min at room temperature in the dark. The protein containing medium was added to stop the reaction and the cells were incubated for another 10 min at room temperature. Flow cytometry analysis was then performed to determine the proliferation based on the fluorescence intensity of the CFSE. The algorithm in FlowJo V. 10.8.01 (BD Bioscience, San Jose, USA) was used to determine the proliferation rate. The stimulation index (SI) was calculated by normalizing the number of proliferated cells from treated immunocompetent skin models to the number of proliferated cells from the untreated control.

### Histology and immunofluorescence staining

The frozen immunocompetent skin models were sectioned (7 µm) using a Leica CM1510 Cryotome (Leica Biosystems, Wetzlar, Germany), subsequently and placed onto Poly-Prep microscope slides (Merck, Darmstadt, Germany). The sections of the immunocompetent skin models were fixed with 4% Roti-Histo Fix (Carl Roth, Karlsruhe, Germany) and subjected to hematoxylin and eosin (H&E) (Carl Roth, Karlsruhe, Germany) or immunofluorescence staining both following standard procedures. For immunofluorescence staining, ImmuSkin M, ImmuSkin MT and ImmuSkin T sections were permeabilized with 0.5% (V/V) Triton X-100 in phosphate-buffered saline (PBS) (both are from Sigma-Aldrich, Munich, Germany) for 15 min. Afterward, the ImmuSkin sections were blocked with 1:20 goat serum (Thermo Fischer Scientific, Karlsruhe, Germany) in PBS for 30 min and incubated with either anti-cytokeratin 10- Alexa Fluor® 647 (Abcam, Cambridge, UK), anti-cytokeratin 14 REAfinity™ (Miltenyi Biotech, Bergisch Gladbach, Germany), anti-loricrin (Abcam, Cambridge, UK), or anti-CD1a (Biolegend, San Jose, USA) overnight at 4 °C. Afterwards, the slides were incubated with a mouse anti-goat Alexa Fluor 488 (Abcam, Cambridge, United Kingdom) or goat anti-rabbit Alexa Fluor 555 (Thermo Fischer Scientific, Karlsruhe, Germany) for 1 h at room temperature. Then, a glass cover slide (Sigma-Aldrich, Munich, Germany) was mounted onto the microscope slide using ProLong™ Gold antifade mountant with 4′,6-diamidino-2-phenylindole (DAPI) (Thermo Fischer Scientific, Karlsruhe, Germany). The ImmuSkin sections were analyzed using a KZ-8001 fluorescence microscope (Keyence, Neu-Isenburg, Germany).

### Statistical analysis

The paired t-tests were used to analyze the comparison of two groups. Statistical significance is indicated as *p-value ≤ 0.05, **p-value ≤ 0.01, ***p-value ≤ 0.001. Treated group responses were analyzed in relation to the untreated group response and the differences between treated and untreated groups were analyzed using Ordinary One-Way ANOVA with Dunnette's correction for multiple comparisons in GraphPad Prism version 9 (GraphPad Software, Boston, USA). The statistical significance of the response (*p*-value ≤ 0.05) is presented by an asterisk (*) above the bar. Data represent means ± standard deviation from 3 independent experiments (cells from three different donors). Post hoc power analysis calculations for the MoLC migration, CD86 expression and the T-cell stimulation index were performed using G*Power version 3.1.9.7 (Faul et al. 2007). Effect sizes for each endpoint were derived from one-way ANOVA results in GraphPad Prism version 9. Eta squared (η^2^) values were calculated using the formula:

$$\eta 2= \frac{{SS}_{between}}{{SS}_{total}}$$

These η^2^ values were then converted to Cohen’s f using:

$$f= \sqrt{\frac{\eta 2}{1-\eta 2}}$$

## Results

### Generation of a multi-layered skin equivalent from hair follicle-derived cells, MoLCs and T-lymphocytes

**Fig. 1:**
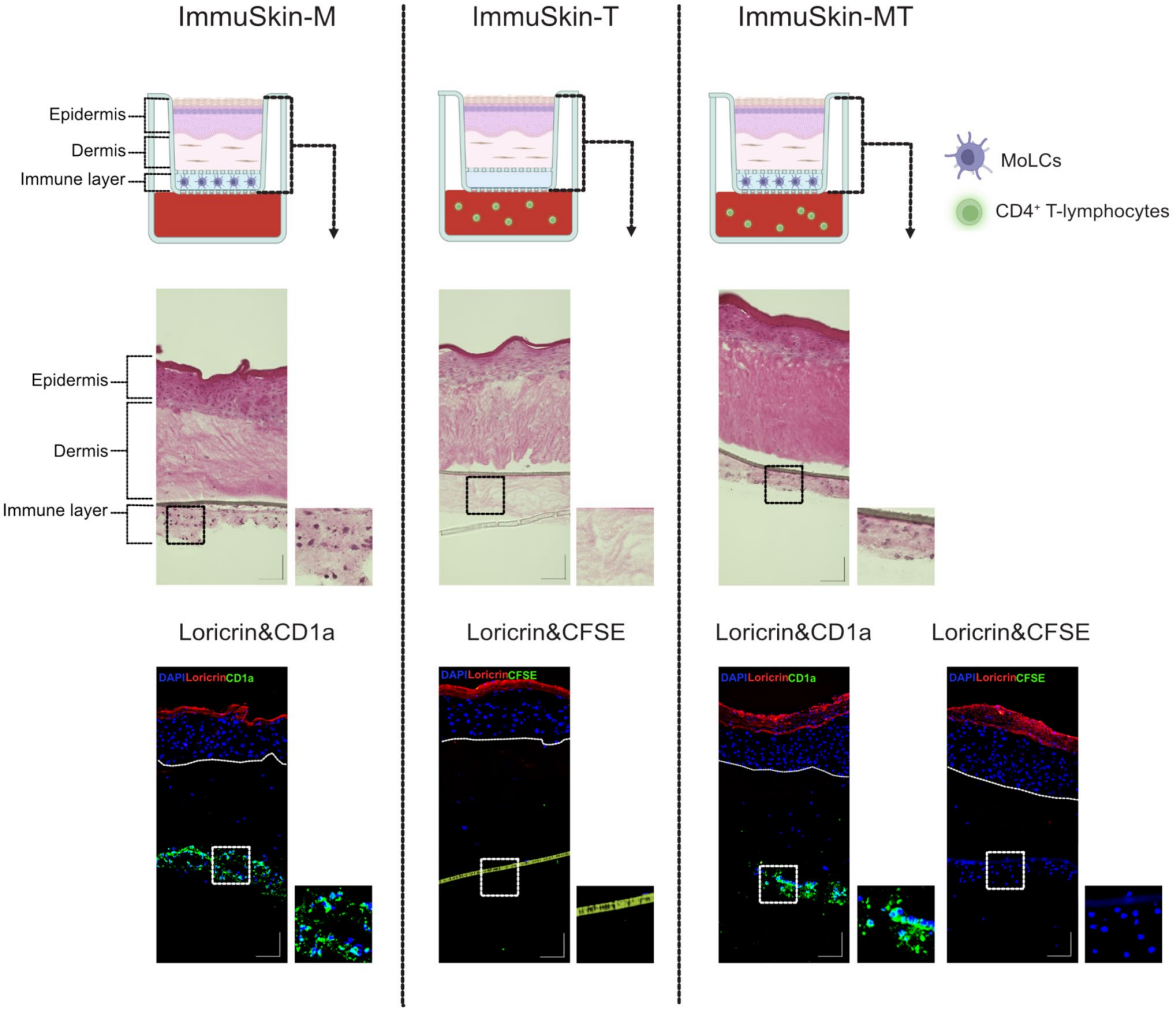
Characterization of ImmuSkin-MT, ImmuSkin-M, and ImmuSkin-T. Skin models were subjected to H&E and immunofluorescence staining against loricrin as a marker for differentiated keratinocytes (red) and CD1a as a specific DC marker (green). DAPI (blue) was used as a nucleus counter-staining. Carboxyfluorescein succinimidyl ester (CFSE, green) is an indicator of T-lymphocytes indicated the absence of T-cells in the epidermis, dermis, and the immune layer. The white dotted line indicates the boundary between epidermis and dermis. In some pictures from immunofluorescence staining, a membrane of the transwell insert is presented in a bright yellow-green strip. Images were captured at a magnification of 20x. Scale bar = 100 µm. n = 3. The schematic part of this figure was created with BioRender.com

The generation of skin equivalents from plucked hair follicles is an easy and non-invasive method which allows easy access to patient-derived cells such as keratinocytes and fibroblasts without taking punch biopsies. Previous studies did not find major differences between skin models generated from skin cells isolated from excised human skin or hair follicles (Löwa et al. 2018). However, due to the lack of immune cells, this model alone has not yet been used to investigate the sensitization potential of chemicals. Based on such an RHS model from hair follicle derived cells, a coculture with MoLCs as well as CD4^+^ T-lymphocytes was developed. This construct was named ImmuSkin-MT and started with the preparation of the RHS. To address the challenge of preventing premature maturation in the coculture of MoLCs, the isolated MoLCs were embedded within a collagen matrix. Then, the RHS was placed on top of the MoLC-containing gel resulting in a three-layer construct composed of the epidermis, the dermis, and the immune layer. This construct was positioned on an insert of a transwell plate containing CFSE-labeled T-lymphocytes in the lower chamber of the transwell plate. This created a coculture construct consisting of HFDK, HFDF, MoLCs and CD4^+^ T-cells, which was also featured by an epidermal barrier structure. Two control constructs were also developed which contained either only MoLCs (ImmuSkin-M) or only CD4^+^ T-lymphocytes (ImmuSkin-T) to study the individual role and interaction of the specific immune cells. ImmuSkin-M was constructed in analogy to ImmuSkin-MT, but without CD4^+^ T-lymphocytes in the transwell chamber. ImmuSkin-T was built with an acellular collagen layer instead of a MoLC layer, whereas T-lymphocytes were placed in the lower chamber of the transwell (Fig. 1). All skin equivalents were characterized by representative microscopic images from H&E staining as well as immunofluorescence staining indicating a comparable appearance of a stratified epidermis and dermis in all three constructs validating the structural integrity of the models (Fig. 1). As expected, MoLCs were only visible in the ImmuSkin-MT as well as the ImmuSkin-M construct with no migration into the epidermal or dermal layer. Naïve CD4^+^ T-lymphocytes, tracked via CFSE labeling, were not detected in the epidermis, dermis and the collagen matrix gel of neither ImmuSkin-T nor ImmuSkin-MT, indicating that they did not migrate from the lower chamber of the transwell into the three-layer construct.

**Fig. 2:**
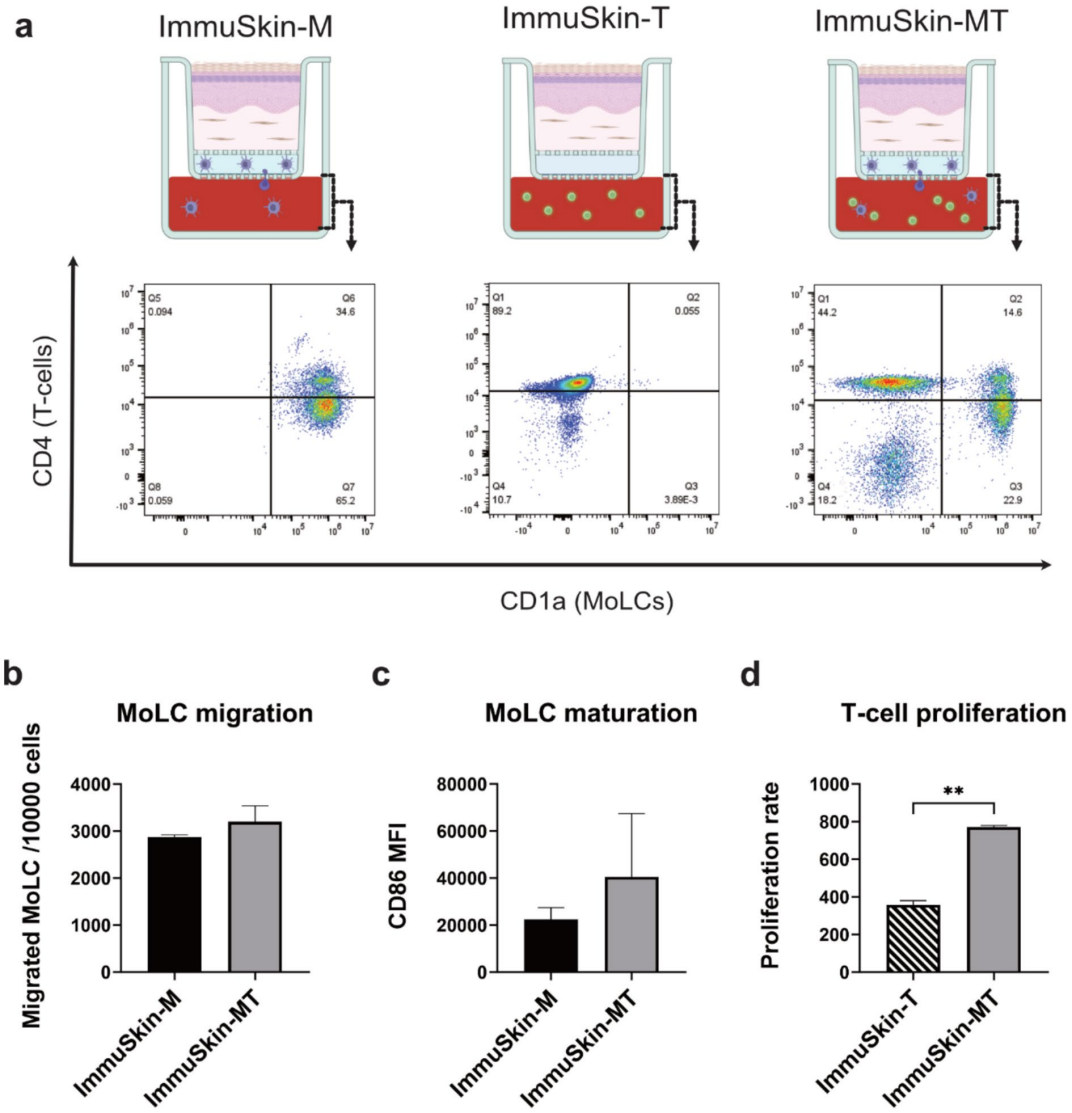
Influence of cocultivation on the migration and maturation of MoLCs and the proliferation of T-cells. Cells harvested from the lower chamber of each ImmuSkin variant were subjected to flow cytometry analysis and stained with anti-CD1a-, anti-CD86- and anti CD4-antibodies. **a** Representative dot plots show the populations of T-lymphocytes and MoLCs from each ImmuSkin type. The upper left side of the quadrant represents T-lymphocytes, while MoLCs are shown in the lower right side of the quadrant. Moreover, MoLC migration (**b**), MoLC maturation (**c**) and T-cell proliferation (**d**) were measured. The black bar represents immune cells from ImmuSkin-M, the striped bar represents immune cells from ImmuSkin-T, while the gray bar represents immune cells from ImmuSkin-MT. Data are presented as mean ± SD, and statistical significance is indicated as *p-value ≤ 0.05, **p-value ≤ 0.01, ***p-value ≤ 0.001. A paired t-test was used for the statistical analysis. *n* = 3. The schematic part of this figure was created with BioRender.com

Next, a flow cytometry analysis was performed to identify different cell types in the transwell chamber of the construct. As expected, CD4^+^ T-cells were detected in the lymphocyte-enriched constructs ImmuSkin-T and ImmuSkin-MT. Interestingly, however, was the fact that MoLCs were also detectable in the lower transwell chamber of ImmuSkin-MT and ImmmuSkin-M indicating that MoLCs can indeed migrate from the collagen matrix into the transwell area. The MoLC were also characterized to determine whether their migration rate or maturation status is altered in the presence of T-lymphocytes. As shown in Fig. 2, the number of CD1a^+^ MoLCs migrating into in the transwell chamber is not increased in the presence of T-cells. Similarly, the expression of CD86 on MoLCs as a maturation marker is not altered when T-cells are added to the construct, indicating that the presence of T-lymphocytes does not influence MoLC maturation and migration. Furthermore, the proliferation of T-lymphocytes was measured in ImmuSkin-T and ImmuSkin-MT constructs. Most interestingly, a significant increase in T-cell proliferation was visible in the presence of MoLCs underscoring the critical role of MoLCs in promoting T-lymphocyte proliferation.

### ImmuSkin-MT as a model for predicting the sensitizing potential of chemicals

**Fig. 3:**
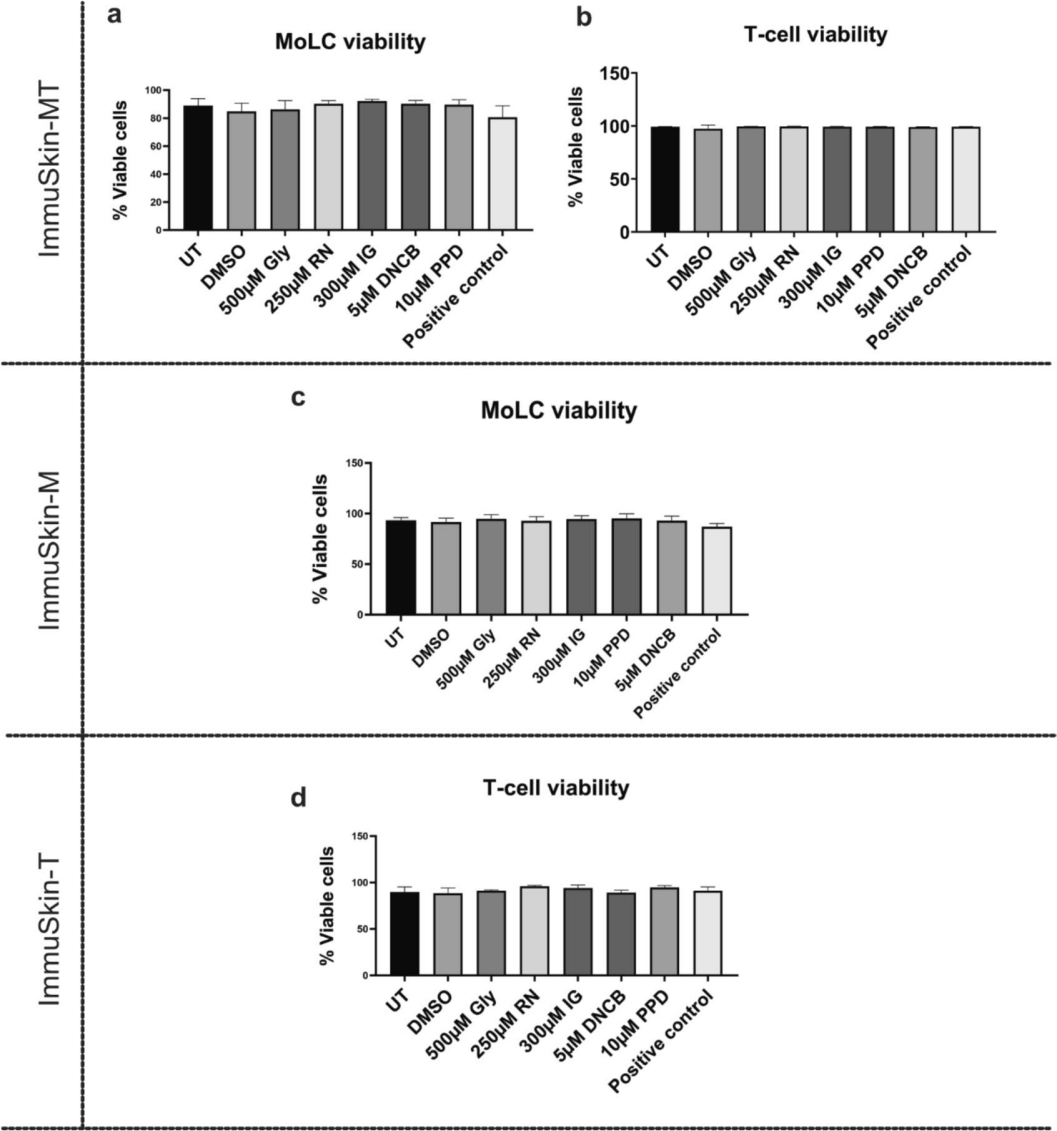
Viability of the cells from ImmuSkin-M, ImmuSkin-T and ImmuSkin-MT. ImmuSkin-M, ImmuSkin-T, and ImmuSkin-MT were topically treated with 0.1% DMSO, 500 μM Glycerol (Gly), 250 μM Resorcinol (RN), 300 μM Isoeugenol (IG), 5 μM 2–4 Dinitrochlorobenzene (DNCB), and 10 μM Paraphenylenediamine (PPD) for 24 h. Cells collected from the lower chamber of each ImmuSkin variant were subjected to flow cytometry analysis and stained with 7-AAD to determine viability. **a** The viability of monocyte-derived Langerhans cells (MoLC) from ImmuSkin-MT. **b** The viability of T-lymphocytes from ImmuSkin-MT. **c** MoLC viability from ImmuSkin-M. **d** The viability of T-lymphocytes from ImmuSkin-T. Statistical significance is indicated as *p-value ≤ 0.05, assessed using Ordinary One-Way ANOVA with Dunnette’s correction for multiple comparisons

Since the ImmuSkin-MT construct mimics the essential features of the skin immune system, such as the dendritic cell layer and the ability to crosstalk between MoLCs and T-lymphocytes, it was investigated whether the model is also able to identify skin sensitizers. To address this, all three constructs were topically treated with known skin sensitizing substances that differed in their potency categorization. After a stimulation period of 24 h, cells of the lower chambers were harvested and subjected to flow cytometry. Examination of cell viability indicated that all used skin sensitizers did not affect the cell viability of either T-cells nor MoLCs in the lower chamber of the transwell. Cell viability was above ≥ 90% across all experimental conditions (Fig. 3).

**Fig. 4:**
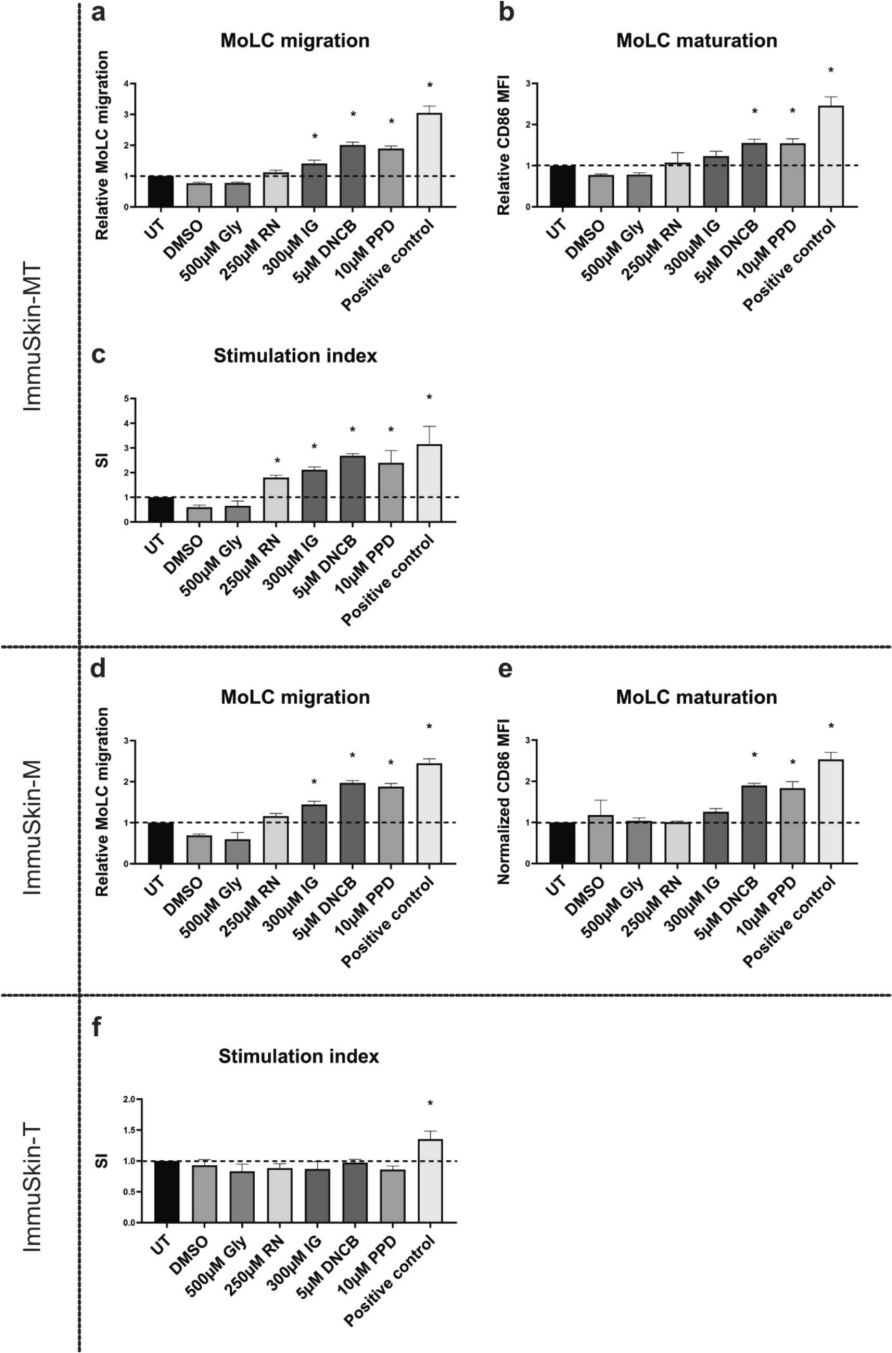
Effect of skin sensitizers on MoLC migration, maturation, and T-lymphocyte proliferation in ImmuSkin constructs. ImmuSkin-M, ImmuSkin-T and ImmuSkin-MT were topically treated with 0.1% DMSO, 500 µM glycerol (Gly), 250 µM resorcinol (RN), 300 µM isoeugenol (IG), 5 µM DNCB, and 10 µM paraphenylenediamine (PPD) for 24 h. Treatment response was assessed by determing MoLC migration and maturation and the stimulation index (SI) of T-lymphocytes. **a** MoLC migration normalized to the untreated control (UT) in ImmuSkin-MT **b** CD86 mean fluorescence intensity (MFI) normalized to the UT in ImmuSkin-MT. **c** SI of T-lymphocytes in ImmuSkin-MT. **d** MoLC migration normalized to UT in ImmuSkin-M. **e** MoLC maturation normalized to UT in ImmuSkin-M. **f** SI of T-lymphocytes in ImmuSkin-T. Data are presented as mean ± SD. Statistical significance is indicated as *p-value ≤ 0.05, assessed using Ordinary One-Way ANOVA with Dunnette’s correction for multiple comparisons. The dotted line represents the baseline. *n* = 3. A post-hoc statistical power analysis revealed that the achieved statistical power for MoLC migration, MoLC maturation, and the SI of ImmuSkin-MT are 0.836, 0.809, and 0.771, respectively

After confirming that the tested skin sensitizers did not exert any cytotoxic effects on MoLCs or T-cells, the migration and maturation of MoLCs were subsequently assessed within the ImmuSkin-MT construct. As expected, the untreated control did not promote MoLC migration or maturation. In contrast, the positive control (TNF-α/IL-1β) induced a threefold increase in migration and 2.4-fold upregulation of CD86 expression. Similarly, the extreme sensitizers DNCB and *p*-phenylenediamine also significantly enhanced both, migration and maturation of MoLCs. In comparison, the moderate sensitizer isoeugenol elicited a moderate response, with a marked increase in cell migration but only a slight enhancement in maturation. The weak sensitizer resorcinol did not stimulate a significant CD86 expression or cell migration (Fig. 4a&b). Similar responses were observed in the ImmuSkin-M construct (Fig. 4d&e). These findings suggest that measuring MoLC migration and maturation primarily allows for the detection of extreme sensitizers. Consequently, it became of particular interest to evaluate T-lymphocyte proliferation within the ImmuSkin-MT construct through the assessment of the stimulation index. As shown in Fig. 4c, the positive control and the strong sensitizers led to an increased proliferation rate of T-lymphocytes 2.6 and 2.4 times for DNCB and *p*-phenylenediamine respectively. Here, also the moderate and weak skin sensitizers isoeugenol and resorcinol induced a significant increase in T-cell proliferation in ImmuSkin-MT. In contrast, the ImmuSkin-T did not show any significant response of T-lymphocytes to the treatments, except after exposure to the positive control, highlighting the importance of MoLCs in in the process of T-cell activation by skin sensitizers (Fig. 4f). In conclusion, these results confirm that ImmuSkin-MT demonstrates a remarkable capacity to faithfully recapitulate the intricate skin sensitization phase observed in physiological skin. Notably, this includes the migration and maturation of LCs, along with the dynamic crosstalk between LCs and T-lymphocytes in response to skin sensitizers. This crosstalk enables also the detection of moderate and weak skin sensitizers. Accompanied by the results from ImmuSkin-M and ImmuSkin-T, the developed immunocompetent skin model ImmuSkin-MT can simulate the immunological response events that occur in physiological skin.

## Discussion

RHS models are a versatile tool that have been applied across various research fields. They represent one of the most promising non-animal alternatives for evaluating the skin sensitization potential of chemical substances (Chau et al. 2013; Schellenberger et al. 2019). However, most existing skin models rely on keratinocytes and fibroblasts derived from juvenile or neonatal foreskin (Matei et al. 2019; Reijnders et al. 2015; Salameh et al. 2021; Schmidt et al. 2020; Zoio et al. 2021).While well-characterized, these models present several limitations, including invasive tissue collection, restriction to male donors, and limited access to donor-matched cells for repeated use.

Hair follicle-derived cells offer a promising solution to these challenges, enabling the generation of RHS models from both male and female donors through minimally invasive procedures (Löwa et al. 2018; Matsuzawa et al. 2019; Schneider et al. 2021). Moreover, they have already been successfully used in disease-specific skin models. For example, personalized RHS models reflecting atopic dermatitis phenotypes have been developed using keratinocytes isolated from the outer root sheath of hair follicles obtained from atopic dermatitis patients (Emmert et al. 2024). This not only highlights the feasibility of using hair follicle-derived cells for disease modeling but also underscores their potential for patient-specific in vitro systems.

Despite these advantages, immunocompetent RHS models derived from hair follicle cells have not yet been developed. This gap likely stems from the technical complexity of co-cultivating skin-resident cells alongside immune cells, which is essential to replicate the intricate immunological microenvironment characteristic of inflammatory skin conditions such as atopic dermatitis. Immune components are equally critical for modeling immunologically driven processes like skin sensitization, in which keratinocytes, dendritic cells, and T cells all play central roles in the initiation and elicitation of allergic responses.

While alternative cell sources and disease modeling capabilities have advanced RHS technology, conventional models remain mechanistically limited in scope. Specifically, they are designed to address only key event 2 (keratinocyte activation) within the AOP of skin sensitization. Their applicability is, therefore, confined to this step, without accounting for earlier events such as hapten-protein binding (key event 1) or downstream immune activation involving dendritic cells and T lymphocytes (key events 3 and 4). The AOP framework proposed by the OECD provides a structured approach to analyze sensitization mechanisms by delineating each contributing event and guides the development of more predictive and comprehensive in vitro models.

Numerous in vitro assays have been developed to assess individual key events in skin sensitization. While several are now included in OECD test guidelines, each captures only one step in the AOP. For instance, assays targeting key event 3 (dendritic cell migration and maturation) have demonstrated robustness (Ouwehand et al. 2008, 2011), though they are not yet guideline-accepted. Even fewer models address key event 4, T-cell activation and proliferation. These are typically used in sensitizer-specific studies such as those involving p-phenylenediamine or Brandowski’s Base, rather than broad screening (Coulter et al., 2010; Moed et al., 2005; Oakes et al., 2017). Among in vivo models, the Local Lymph Node Assay (LLNA) remains a benchmark, quantifying murine T-cell proliferation in response to sensitizers (Gerberick et al., 2007). However, similar to their in vitro counterparts, these models generally represent only a single key event, thereby limiting their utility in capturing the full complexity of the sensitization process.

One of the primary challenges in advancing these models lies in the co-culture of keratinocytes with immune cells, which is critical for reproducing the cellular interplay of the skin's immune environment. Spatial separation strategies, such as embedding immune cells within an extracellular matrix compartment, have been explored to mitigate cell–cell contact issues. Though not entirely physiological, such setups have been shown to support functional intercellular communication (Sapudom et al. 2020), suggesting viable pathways toward multi-event sensitization modeling.

Several RHS models have been developed that integrate either DCs or T-lymphocytes. For example, monocyte-derived DCs have been embedded in agarose-fibronectin gels positioned between dermal and epidermal layers (Chau et al. 2013), and others have incorporated monocyte-derived DCs or THP-1 cells directly into RHS constructs (Bock et al. 2018; Böttcher et al. 2025; Hölken et al. 2023). Models including T-lymphocytes have also demonstrated immune functionality (Kühbacher et al. 2017; Shin et al. 2020; Wallmeyer et al. 2017). However, to date, no model has successfully incorporated both DCs and T-cells in a single system. Achieving this would represent a crucial step toward establishing fully immunocompetent models capable of reproducing the entire cascade of skin sensitization.

To address this, a full-thickness RHS model was developed using only primary human cells, including fibroblasts and keratinocytes isolated from hair follicles of both male and female donors via a minimally invasive method. MoLCs and naïve T-lymphocytes were isolated from buffy coats and integrated into the model. The resulting HFDF and HFDK populations underwent phenotypic characterization and were compared to foreskin-derived cells. Flow cytometry confirmed similar expression profiles, fibroblasts from both sources expressed CD10, CD44, and CD90, while keratinocytes showed consistent levels of CK5, CD29, and CD49f (Supplementary Fig. S1).

Four model variants, RHS, ImmuSkin-MT, ImmuSkin-M, and ImmuSkin-T, were established using hair follicle-derived cells. All models were evaluated through H&E staining and immunostaining for epidermal differentiation markers Loricrin, CK10, and CK14. The HFDKs formed a stratified epidermis with a fully differentiated outermost layer, while fibroblasts were embedded within the dermal collagen matrix. Epidermal marker expression was consistent across all ImmuSkin variants and the RHS control. CD1a + MoLCs were detected only in ImmuSkin-M and ImmuSkin-MT, whereas T-lymphocytes were undetectable under baseline conditions in all models, showing that they remained in the transwell and did not migrate into the skin RHS. Importantly, the presence of immune cells did not alter the keratinocyte differentiation process, as the expression and localization of key epidermal markers remained unaffected (Supplementary Fig. S2). Compared with normal skin, our ImmuSkin constructs differ in that the MoLCs are not placed within the epidermis but underneath the fibroblasts. Thus, it was of great interest, to examine whether this layering affects the immune response.

Our data indicate, that MoLCs within ImmuSkin-M and ImmuSkin-MT migrated from the RHS into the transwell and expressed the co-stimulatory molecule CD86, even in the absence of stimulation, indicating a baseline level of maturation. Upon exposure to moderate or strong skin sensitizers, MoLCs exhibited significantly increased migration and maturation, suggesting a robust immune response to the sensitizing compounds. These findings are consistent with previous studies that have used Langerhans cell migration as a readout for skin sensitizer exposure (Azam et al. 2006; Bock et al. 2018; Gibbs et al. 2013; Jacobs et al. 2006; Rees et al. 2011a; Villablanca & Mora 2008).

Furthermore, the findings from ImmuSkin-T and ImmuSkin-MT highlight the critical importance of studying the interactions between MoLCs and T-lymphocytes, or T-lymphocytes alone, in the identification of skin sensitizers. Specifically, T-lymphocytes in the ImmuSkin-T model exhibited limited responsiveness to all substances except the strong positive control. In contrast, T-lymphocytes in the ImmuSkin-MT model responded robustly to all tested sensitizers. These observations align with previous studies in the field of skin sensitization (Bennett et al. 2007; Betts et al. 2017; Hunger et al. 2004; Lee et al. 2012; Peiser et al. 2004). A key advantage of integrating T-cells into the skin model system is the enhanced sensitivity of the assay. Unlike models containing only MoLCs, which predominantly detect extreme sensitizers such as DNCB and *p*-phenylenediamine, the addition of T-cells enables the detection of moderate and weak sensitizers as well, significantly broadening the assay's applicability.

According to the classification system defined by the European Centre for Ecotoxicology and Toxicology of Chemicals (ECETOC) in the Technical Report No. 87 Contact Sensitisation: Classification According to Potency, skin sensitizers are categorized as extreme, strong/moderate, or weak based on human data such as epidemiological evidence or patch tests, and validated animal assay (ECETOC 2003). Within this framework, DNCB and p-phenylenediamine are considered extreme sensitizers, isoeugenol as moderate, and resorcinol as weak. Glycerol, lacking any sensitization potential, served as a negative control in this study, alongside DMSO as the vehicle (D. Basketter 2021; D. A. Basketter & Scholes 1992; G. F. Gerberick et al. 2004). Given their established potency classifications, these substances were strategically selected to evaluate the discriminatory capacity of the immunocompetent skin model. While OECD reference chemical sets typically include 10–20 substances for comprehensive validation, the present study was designed as a proof-of-concept to assess the model’s biological responsiveness and ability to detect skin sensitization. The selected panel represents a broad spectrum of sensitization potency, from extreme sensitizers to non-sensitizers, and capturing both chemical and mechanistic diversity. All six substances are included in Annex 2 of the OECD Test Guideline 497 supplementary data, underscoring their relevance and frequent use in method development and validation (OECD 2023b). This focused panel was therefore considered appropriate for initial assessment.. The immunocompetent skin models used in this study demonstrated the capacity to distinguish these reference compounds in accordance with their known sensitization potency.

Although limited in number, the tested substances allow for the proposal of a preliminary classification scheme based on key immunological responses. Substances that induce strong MoLC migration, significantly increased CD86 expression, and robust T-cell proliferation may be categorized as extreme sensitizers, as demonstrated by DNCB and *p*-phenylenediamine. Moderate sensitizers, such as isoeugenol, stimulate MoLC migration and T-cell proliferation but lacks a significant increase in CD86 expression. In contrast, substances like resorcinol, which stimulate T-cell proliferation without affecting MoLC maturation or migration, may be classified as weak sensitizers. Importantly, only the negative control substances, DMSO and glycerol, failed to induce any of these immunological markers, further validating the assay’s specificity.

In the context of regulatory testing strategies, the ImmuSkin-MT model may offer significant value as a complementary assay to current OECD-accepted methods. Notably, the OECD Test Guideline 497 (OECD 2023a) outlines a Defined Approach for skin sensitization based on data integration from three in vitro assays (DPRA, KeratinoSensTM, and h-CLAT), each addressing one of the first three key events of the AOP of skin sensitization. However, none of these assays directly capture key event 4, T-lymphocyte activation and proliferation. By integrating both MoLCs and naïve T-cells, the ImmuSkin-MT model uniquely addresses key event 3 and 4 simultaneously, thereby expanding the mechanistic coverage of sensitization testing. With further validation, including reproducibility assessments and an expanded panel of OECD reference substances, the model could evolve into a valuable component of an advanced Defined Approach framework, enhancing both predictive performance and mechanistic relevance for regulatory decision-making. To enable regulatory acceptance, the ImmuSkin-MT model would need to undergo a formal validation process in line with the OECD Guidance Document No.34 (OECD 2005), which outlines the requirements for assessing the reliability, reproducibility, and relevance of new in vitro methods. This includes intra- and inter-laboratory reproducibility studies, expansion of the chemical reference set of the skin sensitizers and development of a standardized and transferable protocol. The formal validation process outlined in this OECD Guidance Document is essential for a new method to be adopted as an OECD Test Guideline and applied for regulatory purposes. Equally important is the implementation of the Good In Vitro Practices (GIVIMP), as described in OECD Guidance Document No.286 (OECD 2018). GIVIMP provides quality assurance guidance to ensure scientific and technical robustness by emphasizing traceability of reagents and cell sources, equipment qualification, trainings, documentation, and predefined acceptance criteria for each experimental step. Recent studies have shown the importance of such quality standards and inter-laboratory benchmarking for the validation of experimental methods such as complex 3D models (Bas et al. 2021; Jacobs et al. 2024). Adoption of these principles will be essential for the progression of ImmuSkin-MT toward integration into a future Defined Approach or inclusion in the OECD Test Guideline framework for skin sensitization testing.

In conclusion, this study presents a novel and immunocompetent 3D skin model that captures key events 3 and 4 of the AOP for skin sensitization. The model features a physiologically relevant epidermis and dermis, integration of MoLCs beneath the dermal layer, and co-cultivation with T-lymphocytes in the lower chamber of a transwell system. This setup closely mimics the native interplay between skin-resident immune cells and T-cells, marking a significant advancement in in vitro toxicology.

Although further validation, including expanded testing across a wider range of known sensitizers is required, the current data position the ImmuSkin-MT model as a promising tool for predictive toxicology. By concurrently reflecting multiple key events of the AOP and incorporating two essential immune cell types for the first time, this model enhances biological relevance and reduces reliance on animal testing. ImmuSkin-MT thus represents a meaningful step forward in developing more accurate, ethical, and human-relevant methods for assessing the sensitizing potential of chemical substances.

## Supplementary Information

Below is the link to the electronic supplementary material.

Supplementary file1 (DOCX 2543 KB)

## Data Availability

Data are available from the corresponding authors upon request.
