# Supplementary figures and images for: A hair-follicle reconstructed in vitro immunocompetent skin model for prediction of the sensitizing potential of chemicals
Source: Arch Toxicol. 2025 Jul 18;99(10):4131–44. doi: 10.1007/s00204-025-04130-z (PMC12454623; doi:10.1007/s00204-025-04130-z)

**Supplementary Fig. S1**


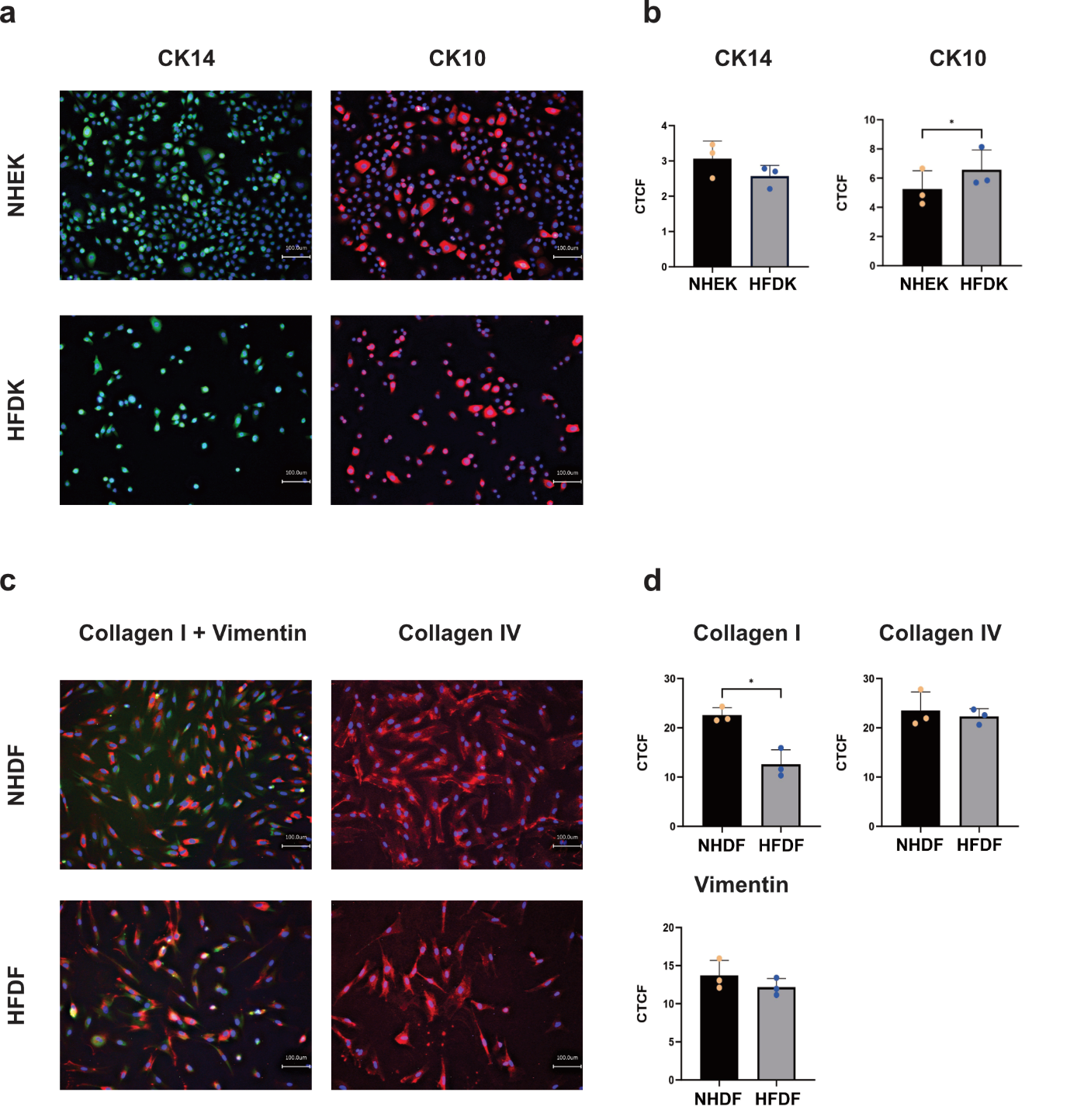


**Supplementary Fig. S2**

**
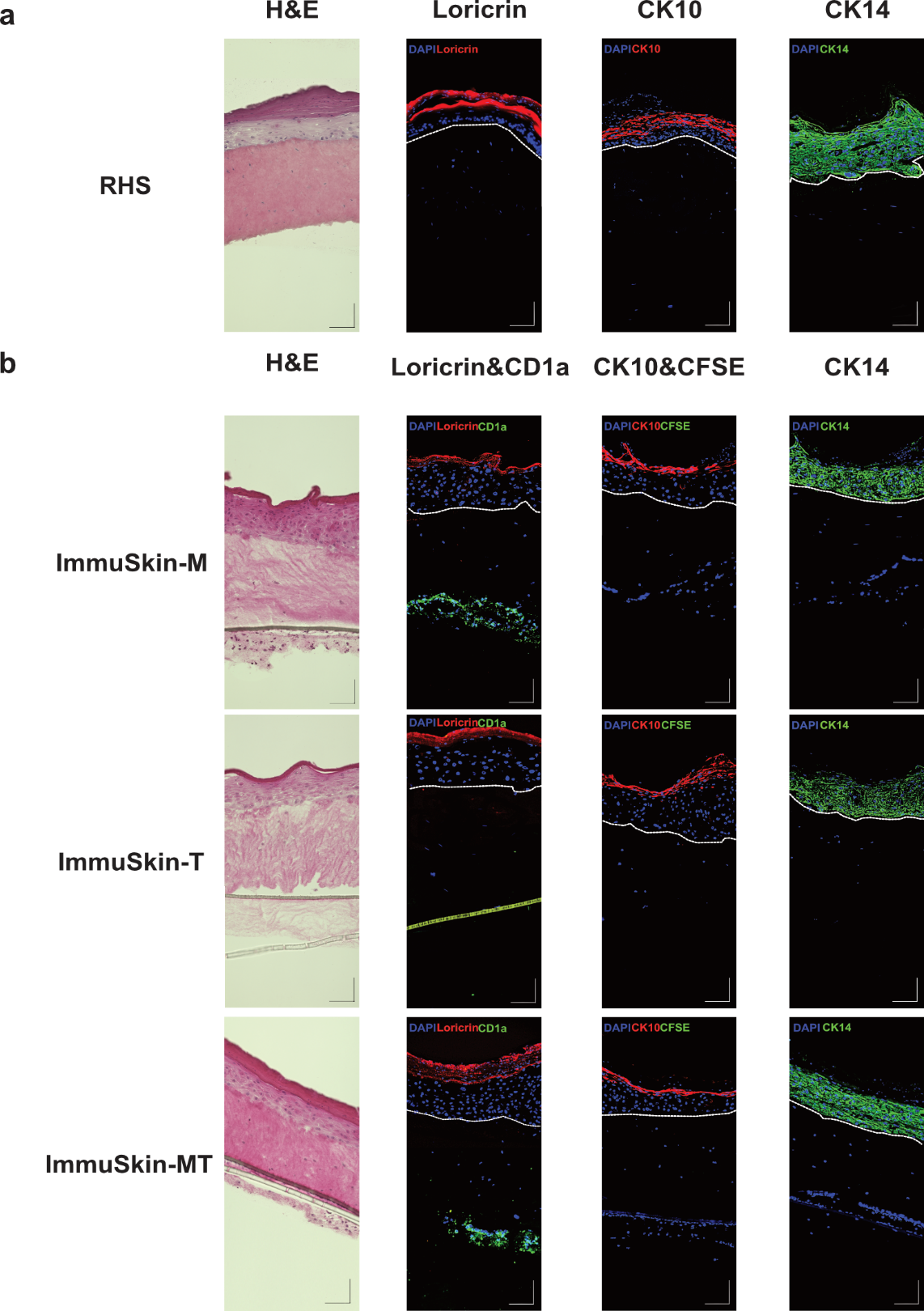
**

Supplement: Supplementary file 1 — Supplementary file1 (DOCX 2543 KB) [file 204_2025_4130_MOESM1_ESM.docx]
